# Mercury Exposure Associated with Use of Skin Lightening Products in Jamaica

**DOI:** 10.5696/2156-9614-10.26.200601

**Published:** 2020-05-04

**Authors:** Phylicia Ricketts, Christopher Knight, Andre Gordon, Ana Boischio, Mitko Voutchkov

**Affiliations:** 1 Department of Physics, Radioecological Lab, The University of the West Indies, Mona Jamaica; 2 Analytical Services Department, Mines and Geology Division, Hope Gardens, Kingston, Jamaica; 3 Pan American Health Organization, Washington DC, USA

**Keywords:** mercury, skin lightening products, exposure assessment, X-ray fluorescence analyzer, Jamaica

## Abstract

**Background.:**

Skin bleaching is a major health concern among Jamaicans. A common ingredient in skin lightening products is mercury. Mercury is a toxic substance that can cause damage to the gastrointestinal tract, nervous system and kidneys.

**Objective.:**

The objectives of this study were to use different analytical techniques to measure mercury concentrations in popular skin lightening products used in Jamaica and to assess individual levels of mercury exposure based on product usage.

**Methods.:**

Sixty skin lightening products were purchased from different vendors across various locations in Jamaica. Each product was initially screened for mercury using a portable handheld energy dispersive X-ray fluorescence (XRF) analyzer. In addition, 25 out of 60 products were further measured using cold vapor atomic absorption spectroscopy (CVAAS). Questionnaires were distributed to users of skin lightening products to determine their usage patterns.

**Results.:**

Six products had mercury concentrations above the United States Food and Drug Administration (FDA) allowable limit of 1 ppm, of which three products contained alarmingly high concentrations (i.e. > 400 ppm). The majority of products (57 out of 60) had mercury concentrations below 10 ppm. The mercury concentrations in skin lightening products ranged from 0.05 ppm to 17,547 ppm. In our sample, 51% of women and 49% of men used skin products more than once per day.

**Conclusions.:**

On average, creams contained more mercury than lotions and soaps. Individuals who use skin lightening products in Jamaica may be at risk for high mercury exposure, as some popular products were found to have mercury concentrations above the allowable limit.

**Competing Interests.:**

The authors declare no competing financial interests.

## Introduction

Skin lightening is the use of chemicals to reduce the amount of melanin in the skin, resulting in a lighter skin complexion.^1^ The skin lightening phenomenon in Jamaica is believed to be a legacy of British colonialism, where there was social discrimination based on skin complexion. Individuals with light colored skin were portrayed as more beautiful and wealthier than persons with dark colored skin. The skin bleaching trend is perpetuated by the influence of mass media and popular culture, which shows light colored skin as an ideal preference of beauty.^2^ Most skin lightening products are only intended to be used for a short period of time, mainly for the gradual fading of dark spots due to acne, freckles and skin conditions such as psoriasis and eczema.

Skin lightening or skin bleaching is a public health concern in countries where these products are popular, such as Jamaica, as skin lightening products may contain toxic substances such as hydroquinone, steroids and mercury.^3^ Mercury exists in three forms: elemental, inorganic and organic. The inorganic form of mercury may be an added ingredient in cosmetics.^4^ However, recent reports have shown that methylmercury is also sometimes added to skin lightening products.^5^ Melanin is responsible for skin color.

Dark-skinned people have more melanin than light-skinned people.^6^ Inorganic mercury is added to skin lightening products to inhibit the formation of melanin. Furthermore, inorganic mercury may be absorbed into the skin through the sweat glands and hair follicles.^7^ The use of products containing inorganic mercury can cause topical damage to the skin, kidneys, and nervous system. Long term exposure to inorganic mercury may also result in irritability, muscle weakness, memory loss and kidney failure.^8^ Pregnant women and women of childbearing age (18–44 years old) are of particular concern regarding mercury exposure because they can transfer mercury to their child. A case report found that a black, Belgian woman who used skin lightening soap containing 1% mercury during pregnancy and lactation recorded high mercury levels in her blood (91 μg/L) and urine (784 μg/g), while her three-month-old infant also recorded very high blood (19 μg/L) and urine (274 μg/g) mercury concentrations.^9^

Previous studies have demonstrated the use of portable handheld X-ray fluorescence (XRF) analyzers to measure mercury concentrations in skin lightening products in cases where mercury concentrations were well above 1000 ppm.^10^,^11^,^3^ The present study investigates the suitability of using portable handheld XRF to measure lower concentrations.

The United States Food and Drug Administration (FDA) has a limit of less than 1 ppm of mercury in skin lightening products. Many European and African nations have banned the use of mercury in skin lightening products. It is suspected that there are some mercury-containing skin lightening products available for purchase in Jamaica. A previous study found that the mercury concentration of one of two skin lightening samples products manufactured in Jamaica was 13,546 times greater than the FDA limit.^3^ Since these mercury-containing products are still available for purchase (both locally and for export), the objective of the present study was to use different analytical techniques to measure mercury concentrations in popular skin lightening products used in Jamaica and to estimate the levels of mercury exposure to users, in order to raise awareness and promote appropriate legislation.

**Abbreviations**
*CVAAS*: Cold vapor atomic absorption spectroscopy
*FDA*: United States Food and Drug Administration
*XRF*: X-ray fluorescence

## Methods

In order to assess mercury exposure from the use of skin lightening products in Jamaica, the project was separated into two phases. The first phase of the project involved collection of data on the types of skin lightening products available for use from twelve (12) locations in Jamaica. Questionnaires were also administered to selected participants to identify skin lightening product usage patterns. The second phase involved measuring the samples for mercury.

### Data collection

**Figure 1:**
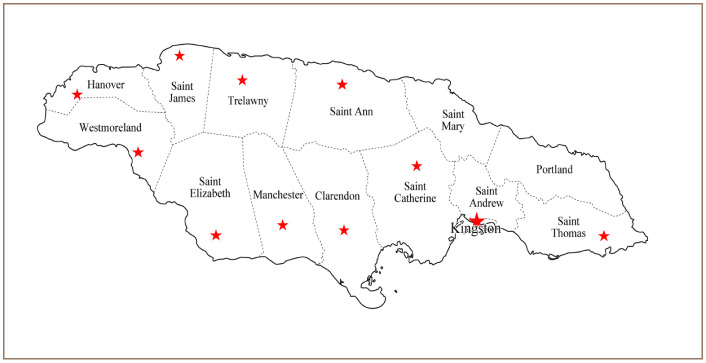
Map showing locations (★) visited during field trips

This study was conducted from February to May 2017. A convenience sample of 384 participants, known to be users of skin lightening products, were invited to complete self-administered questionnaires about their use of skin lightening products (such as skin brighteners, fading cream, scar removal cream, anti-wrinkle cream, etc.). Potential participants were pre-screened to determine if they used skin lightening products. In recruiting the participants, inclusion criteria were males and females who regularly used skin lightening products living in different parishes in Jamaica *(Figure 1).* The questionnaire was also designed to capture information such as gender, age, and side effects of using the products. Participants provided informed verbal consent prior to completing the questionnaire. The questionnaire is included in Supplemental Material 1.

### Sample collection

The research team visited 12 vendors to conduct a market survey to determine the number of skin lightening products available in Jamaica. This survey was similar to the brand survey conducted by the Zero Mercury Working group.^10^ Sixty of the most popular skin lightening products were purchased from different vendors, one from each brand. The name, physical description (such as color and texture) and the main ingredients listed on the labels for each skin lightening product were recorded. A description of each of the products purchased can be found in Supplemental Material 2.

### Analytical techniques

This study used two analytical techniques (X-ray fluorescence (XRF) and cold vapor atomic absorption spectroscopy (CVAAS)) to measure the mercury concentrations in the samples. However, the primary research tool was the XRF analyzer. We wanted to investigate the capability of portable handheld XRF analyzers to detect mercury. All of the samples were initially screened for mercury using a portable handheld NITON XRF analyzer. For quality control purposes, subsamples (25 out of 60) were then analyzed by both techniques and the results compared.

### X-ray fluorescence analysis

The Niton XL3T GOLDD+ portable handheld XRF analyzer was used to measure mercury concentrations in 60 skin lightening products. Each sample was prepared and measured for one minute, in triplicates. The instrument's built-in ‘plastics mode' was used to detect mercury in the samples. The products were categorized based on sample matrix, i.e. creams, gels, lotions and bar soaps. Approximately 10 ml of each product were placed in sample cups and covered with Mylar films, while 5 g of bar soap was cut and measured.

For the recovery experiment, 5 ml of mercury chloride standard, containing 20 ppm of mercury was added to 10 g of a sample (La Bamakoise Tamarin Lait Extra Tonique (lotion)) in the sample cup. The solution was mixed thoroughly and covered with Mylar film. No emulsifiers were used to combine the mixture and only liquid mercury standards were available at the time for calibration purposes. The procedures were repeated with standard solutions containing 40 ppm and 100 ppm of mercury, respectively. The experiment was used to test the accuracy of the analyzer in measuring mercury in this sample matrix i.e. lotion. The mercury recovery in the sample was found to be about 65%. The detection limit of the instrument was calculated as 10 ppm.

### Cold vapor atomic absorption spectroscopy

Cold vapor atomic absorption spectroscopy was used to measure a batch of samples that were below the detection limit of the XRF analysis (10 ppm). The equipment used to measure mercury (Hg) concentration was the Buck Scientific 400A mercury analyzer. The choice of reagents and digestion procedures were similar to those used in previous literature.^12^–^14^ All reagents were of analytical grade. Working standards were prepared by dilution of a 1000 mg/L ascorbic acid (AA) standard stock solution and intermediate standards. Standards covered two ranges: low 0.05 – 0.5 μg Hg/ml and high 1.0 – 5.0 μg Hg/ml. For each sample, about 0.25 g was digested with sulphuric acid and (1:1) perchloric and nitric acid.

The digested solution was made up to 100 ml with distilled water. Fifty (50) ml of digested solution was transferred to a biological oxygen demand bottle used for mercury analysis. Five (5) ml of stannous chloride was added to each biological oxygen demand bottle which was immediately attached to the aeration apparatus. The maximum mercury absorbance after 2 minutes was recorded. For every batch of samples, a blank, a standard, and spike samples were included. A calibration curve for mercury standard and instrument reading was obtained. The detection limit was found to be 0.03 μg/g.

### Quality control and calibration

**Figure 2:**
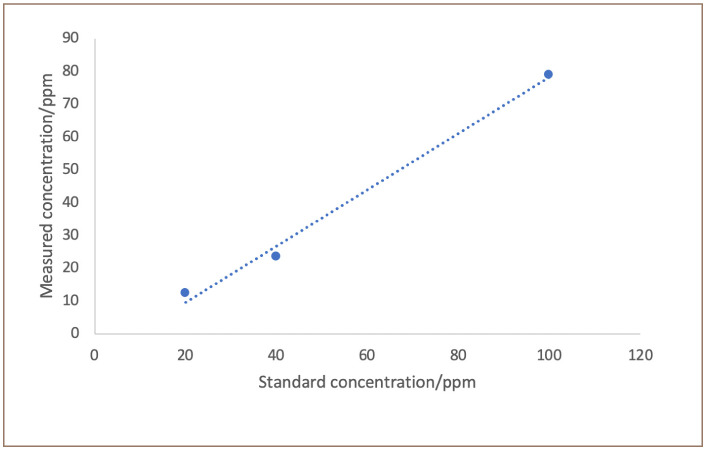
Correlation between samples spiked with mercury measured by portable XRF analyzer and mercury known standard concentrations. R^2^ = 0.99

The accuracy of the XRF analyzer was tested using a multi-element reference material, Niton PN 180–554; batch SN: PE-079-N (ThermoFisher Scientific, Massachusetts, USA). The reference value was 1002 ppm, while the measured value was 969 ± 4.5 ppm. The manufactured settings for handheld XRF analyzer do not include a mercury calibration for these type of sample matrices (i.e. creams and soaps). Therefore, the analyzer was manually calibrated using three samples each spiked with known mercury concentrations. The results *(Figure 2)* were used to create a calibration curve for measuring mercury concentrations in creams and soaps using XRF.

The mercury standards were analyzed in triplicates. The measured values for mercury were consistently lower than the reference standard values. The recovery for mercury in the sample was greater at high concentrations. Using the calibration curve, the estimated value for the instrument detection limit was approximately 10 ppm.

### Statistics

Data analysis was performed using Microsoft Excel 2013, and the Statistical Package for the Social Sciences (SPSS) software program version 17.

## Results

Out of a total of 384 subjects, 196 were female and 188 were male. A large proportion (280 respondents) were younger than 30 years old.

**Figure 3:**
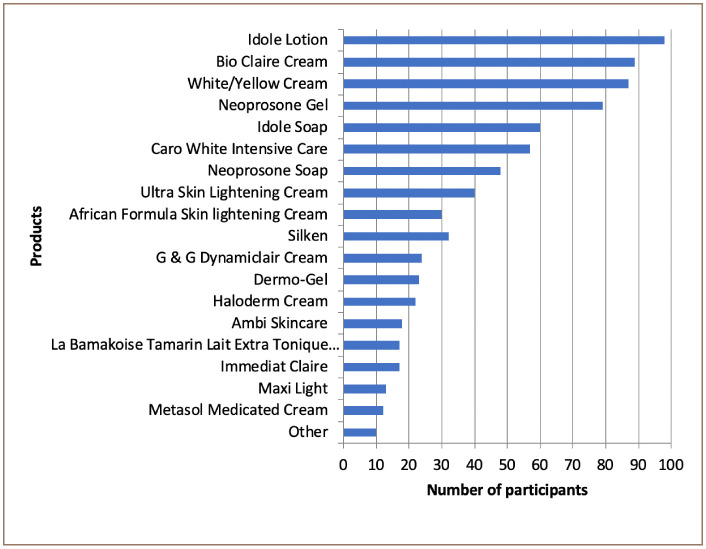
Frequency of specific products reported by respondents (n = 384)

Figure 3 shows the most popular skin lightening products used by participants. Products that were used by less than 1% of participants were categorized as ‘others', including Dolly soap, Crusader Soap and Neoplus Cream Fort. A total of 221 respondents reported mixing different combinations of skin lightening products. All mixed products contain one or all of the following products: Idole Lotion, Neoprosone Gel, Caro White Intensive Care and white/yellow creams.

Table 2 shows that about 53% of skin lightening products purchased in Jamaica were imported from the Ivory Coast, European Union and Lebanon. Some products originating from European nations and the Ivory Coast had mercury concentrations greater than 1 ppm.

Table 1 presents demographic information on study participants. The results showed that the majority of respondents applied skin lightening products more than once per day.

**Table 1:** Demographics of Study Participants

**Demography**	**Number of subjects**
Age groups/years	
<30	280
31–50	92
>50	12
Frequency of application	
More than once per day	197
Once per day	130
Once per week	57
Socio-economic status	
Employed	192
Unemployed	84
Student	108

**Table 2:** Country of Origin of Skin Lightening Products Purchased in Jamaica (n=109)

**Country of origin**	**Number of products (%)**
Ivory Coast	19
European Union	19
Lebanon	15
United States	15
England	10
Jamaica	8
India	8
Other	6

**Figure 4:**
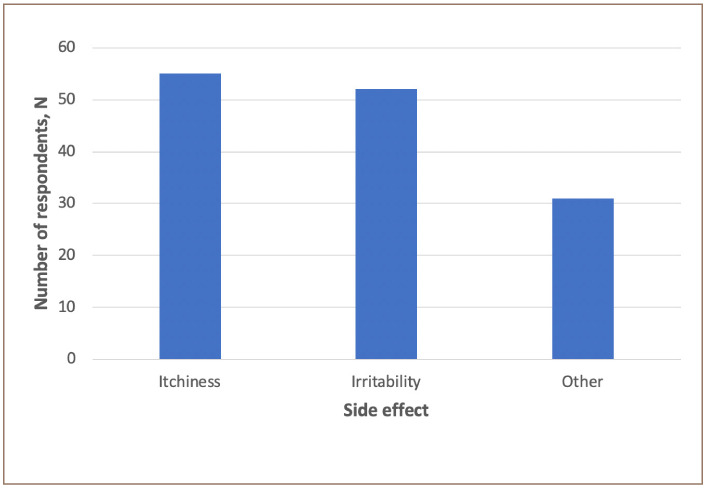
Frequency of reported health effects for the use of skin lightening products

Figure 4 summarizes the health effects reported by respondents. The most frequently reported health effects were dermal irritability, itchiness and ‘other' – headaches, scars and depression. Thirty subjects reported headaches, scars and depression, and 93% of these subjects used products containing more than 1 ppm of mercury.

### Analytical data

Supplemental Material 3 shows the mercury concentrations of products (total of 60 products) measured by the handheld XRF analyzer. The majority of products (95%) were below the instrument detection limit (10 ppm). The measured products ranged from 0.05 to 17,547 ppm. The samples were placed in three categories < 10 ppm, 10 – 1000 ppm, and >1000 ppm. Fifty-seven samples had mercury concentrations below 10 ppm, while 3 out of 60 products contained alarmingly high concentrations of inorganic mercury (>400 ppm).

Twenty-five (25) samples with mercury concentrations below the XRF detection limit were selected and analyzed by CVAAS and the results are shown in Table 3. Mercury concentrations ranged from 0.05 ppm to 3.68 ppm. In Table 3, products with the mean lowest mercury concentrations (number = 9) were placed in the lower quartile, while products with the mean highest mercury concentrations (number = 6) were placed in the upper quartile.

**Table 3:** Mercury Concentrations of Products Measured Using Cold Vapor Atomic Absorption Spectroscopy (n = 25)

**Quartile**	**Mean Hg concentration (ppm ± SD (n))**	**Hg range**	**Name of products**
1	0.06 ± 0.02 (n = 9)	0.05 – 0.10	Bio Claire Oil Bio Claire Soap BioTone Caro Bright Fast Action Crusader Soap Glow and White Hyprogel La Bamakoise Tamarin Lait Extra Tonique (lotion) Radiant Skin Lightening Pills
2	0.14 ± 0.02 (n = 5)	0.12 – 0.16	7-Day Magic Lightening Cream Bio Claire Lotion Caro White Intensive Care Idole Soap Neoplus Soap Fort
3	0.24 ± 0.05 (n = 5)	0.18 – 0.29	G&G Dynamiclair Cream Metasol Medicated Cream Natural Papaya Cream Tamarind Lightening Cream Ultra Bright Cleansing Bar
4	1.41 ± 1.3 (n = 6)	0.33 – 3.68	African Formula Skin Lightening Cream Bio Claire Cream Dolly Antiseptic Soap Haloderm Cream KomeFast Super Toning Cream Maxi Light

Abbreviations: Hg, mercury; SD, standard deviation; n, number.

See Supplementary Material for a list of products and mercury concentrations.

Table 4 shows that mercury concentrations varied based on the product formulations. The highest mercury concentrations were found in cream formulations.

**Table 4:** Mercury Concentrations in Skin Lightening Products (n = 25) Based on Product Formulation

**Product category**	**Mean ± SD (ppm)**	**Range (ppm)**
Soap (n = 7)	0.18 ± 0.11	0.05 – 0.45
Cream (n = 14)	0.67 ± 1.04	0.05 – 3.68
Lotion (n = 2)	0.11 ± 0.01	0.08 – 0.12
*Other (n = 2)	0.05 ± 0.01	0.05 – 0.06

*includes Bio Claire Oil and skin lightening pills.

Abbreviations: SD, standard deviation; n, number.

## Discussion

Skin lightening or bleaching is a major public health concern in Jamaica. Many people ‘lighten' their skin for a perceived improvement in appearance, status or wealth. There have been many efforts to raise awareness of the possible risks posed by these skin lightening products in an attempt to lower their rate of use.^4^

Combining the use of skin lightening products in Jamaica is a common practice. Many users purchase and mix different products at home in an attempt to improve potency. The most common products used in homemade mixtures were ‘white cream' and ‘yellow cream'. These products have mercury concentrations above 400 ppm, therefore increasing the risk of high mercury exposure. It is generally advised that persons should not mix skincare ingredients because it may cause irritation or allergic reactions.^15^

The FDA has set an allowable limit of 1 ppm of mercury in skin lightening products.^4^ In the present study, the results from the CVAAS analysis showed that 3 out of 25 products tested contained mercury concentrations greater than 1 ppm. Meanwhile, the XRF analysis showed that 3 out of 60 products recorded mercury concentrations above 400 ppm, with the highest observed value of 17,547 ppm. Interestingly, only one product listed mercury as an ingredient on its label. Products with mercury concentrations above 400 ppm were reported to be used by at least 50% of respondents.

Some skin lightening products used in Jamaica are imported from other countries. Different regulatory bodies have set limits of 1–3 ppm of mercury allowed in non-eye area cosmetics.^4^ The European Union and many African nations have banned the use of mercury in all cosmetics.^4^ However, results from the present study found some products from European nations and the Ivory Coast had mercury concentrations greater than 1 ppm. Hamann *et al.* measured mercury content in skin lightening products from different countries in 2014 and found that products from Thailand (mean ± SD 45,622 ± 322 ppm) had the highest concentrations, followed by China (38,535 ± 298 ppm), Jamaica (13,546 ± 607 ppm) and Japan (10, 753 ± 124 ppm).^3^ Other studies showed that a skin lightening product manufactured in Jamaica had a concentration of up to 14 000 ppm.^16^

Most skin lightening products purchased from registered pharmacies had mercury concentrations within the FDA allowable limit of 1 ppm.

There were two unlabeled packages with mercury concentrations greater than 400 ppm. These products were identified as ‘white cream' and ‘yellow cream'. Information regarding their country of manufacture and commercial names were not available.

Levels of mercury exposure can be estimated based on the concentrations of mercury in products and usage patterns. Table 5 shows the range of mercury concentrations in products used in different countries. The results were obtained from an analysis of previous studies that measured mercury concentrations in the most popular skin lightening creams across countries. It is estimated that individuals living in Jamaica, Mexico and the US may be exposed to very high levels of mercury in skin lightening products. Therefore, it is important to monitor products originated or distributed in these countries

**Table 5:** Mercury Concentrations in Popular Skin Lightening Products Across Countries

**Country**	Hg concentration range* (ppm)	**Reference**
Jamaica	0.05–17345.00	Current study
Ghana	0.006–0.549	17
Malaysia	0.00–1.13	18
India	0.14–0.36	19
Mexico	878.00–36000.00	20
China	0.00–0.59	21
United States	1729.00–45622.00	3
West Africa	0.00005–0.27	22

*The number of significant figures varied according to reference articles

Abbreviation: Hg, mercury

When a skin lightening product is applied to the skin, the mercury is most likely absorbed through the skin. Some of the factors that may influence dermal absorption include hydration of the outer layer of the skin and the frequency of application.^22^ In our sample from Jamaica, 48% of respondents applied the products more than once per day, thereby increasing their dermal absorption. Other factors that affect the rate of mercury absorption are skin thickness and external temperature. Individuals who ‘lighten' their skin will generally apply the product to their face. In applying skin lightening products to the face, individuals may absorb 5–10 times more mercury in their blood than if products are applied to other parts of the body, as skin tissue of the face and scalp is relatively thinner.^23^ Age could also impact skin thickness, as children have thinner skin than adults. Therefore, children are likely to absorb more mercury if they use skin lightening products. A previous case in California showed that infants displayed elevated levels of mercury in urine when exposed to a product being used by the entire household.^24^

In the present study, the majority of skin lightening product users were females younger than 30 years of age. This particular group is of particular concern in terms of mercury exposure because they are of reproductive age. Previous studies have shown cases of inorganic mercury found in the placenta.^25^ After pregnancy, the levels of inorganic mercury in both infant's and mother's blood have been found to be the same.^26^ Some studies have connected the influence of temperature in the environment and the risk of mercury absorption. For temperatures ranging from 37^°^C to 50^°^C there was a two-fold increase in skin permeability. Therefore, for persons living in tropical climates, the rate of absorption for mercury compounds in skin-lightening products is greater than that for those living in cool climatic regions,^27^ highlighting another risk factor for the population in Jamaica with regard to these products.

Product category may also influence mercury exposure. Table 4 shows the mercury concentrations in skin lightening products based on product category. Our results showed that cream formulations had the highest concentrations of mercury, while products with lotion formulations had the lowest concentration of mercury. Many (38%) of the skin lightening products used in the present study were aqueous formulations such as creams. Previous studies have reported cases of the health effects of mercury exposure from the use of skin lightening products. Individuals exposed to 1000 ppm to 38,000 ppm of mercury displayed symptoms of mercury poisoning and ultimately kidney damage, while persons exposed to mercury less than 25 ppm did not display any critical symptoms.^9^ Although there were some products in Jamaica containing more than 1000 ppm of mercury, to our knowledge, there have been no reported cases of mercury poisoning as a result of using skin lightening products. The main reported side effects were itchiness and irritability. The results showed that the number of reported side effects were slightly greater among those who used products with mercury concentrations higher than 1 ppm. There were 138 cases of reported side effects and 79 cases involved individuals who used products with mercury concentrations higher than 1 ppm. These side effects are common symptoms of mercury exposure, however, they may also be related to other external factors, such as allergens. There have been limited published data on the health effects for individuals exposed to low levels of mercury from the use of skin lightening creams.

### Risk communication

A series of public awareness campaigns were conducted with these research results. These included oral and poster presentations at the ‘Dying to be Beautiful' conference in December 2017 and the Ministry of Health Jamaica—Annual Health Research Conference in November 2018. Flyers and brochures on the health risks of mercury exposure were also designed and distributed to university students and the general public who attended the workshops and expos. Based on our analysis of the ingredients listed in these skin lightening products, users were advised to avoid products with various names of mercury, such as *mercuric iodide, mercurous chloride, ammoniated mercury, amide chloride of mercury, quicksilver, cinnabaris (*mercury sulfide*), hydrargyri oxydum rubrum (*mercury oxide*)* and *calomel.* These campaigns targeted healthcare professionals and members of vulnerable groups such as females under 30 years of age.

### Further recommendations

The present research was carried out to support tasks under Minamata Convention Articles 4 and 16, which encourages parties to identify populations at risk, raise public awareness of mercury exposure and transfer this knowledge. Based on the observed results, further risk assessment is recommended to determine potential mercury exposures. The results of this study provide qualitative information on dermally absorbed mercury. The ideal biomarker for inorganic mercury exposure from skin lightening products would be a urine sample, however no biomarkers were used in the present study. It is suggested that further research be conducted to quantify mercury concentrations in urine and investigate relationships with usage patterns for skin lightening products. Some of the products with higher mercury concentrations were imported, and therefore, there is a need for stronger efforts to ensure compliance with appropriate legislation. The product with the highest mercury concentration was manufactured and distributed in Jamaica and has mercury clearly listed in the ingredient list. In this case, the public should be educated about the risks of using these products. In addition, continuous public awareness is needed in order to prevent exposures among vulnerable populations such as pregnant women and children.

## Conclusions

The results of the present study provide an overview of potential mercury exposure in Jamaica. Six skin lightening products were found with mercury concentrations above the FDA allowable limit of 1 ppm. Based on the data obtained on product usage patterns, some Jamaicans may be at high risk for mercury exposure due to popular products having mercury concentrations up to 17,000 ppm. External factors were also noted to affect mercury exposures, such as combining mercury-containing products that contribute to mercury absorption in this particular demographic. The results from this study will aid direct consideration under the Minamata Convention, Annex A of Article 3, and other health considerations, under Article 16, 17 and 18 on health, in addition to the furthering of information and research on human exposure to mercury.
