# Supplementary material for: Mercury Exposure Associated with Use of Skin Lightening Products in Jamaica
Source: J Health Pollut. 2020 May 4;10(26):200601. doi: 10.5696/2156-9614-10.26.200601 (PMC7269324; doi:10.5696/2156-9614-10.26.200601)
Supplement: Supplementary file 1 [file Ricketts_Supplemental1.docx]

**Supplemental Material 1**

**Questionnaire**

**Internal use**

Date:

Sample ID:

**Assessment of skin whiteners/skin brighteners/fading creams/bleaching
creams usage in Jamaica**

1. Gender?

Male Female

1. Age group?

Under 30 years old 31 – 50 years old Over 50 years old

1. What is your current employment status?

Employed Unemployed Student

1. How often do you use skin whiteners/skin brighteners/fading cream/bleaching creams?

Once per day More than once per day Once per week

1. How long have you been using skin whiteners/skin brighteners/fading cream/bleaching cream?

Less than 3 years More than 3 years

1. Name (List) all the skin care products you use.

_____________________________________________________________________________________________________________________________________________________________________________________________________________________________________________________________________

1. Do you use mixtures of skin whiteners/skin brighteners/fading cream/bleaching cream? If Yes, what are the combinations?

_______________________________________________________________________________________

1. Do you notice any negative effects while using these products? (Select more than one if necessary)

Discoloration Itchiness Irritability Depression Other:________

1. Do you have any skin care product that you would like to test for mercury? Leave us your contact information.
