# Supplementary material for: Mercury Exposure Associated with Use of Skin Lightening Products in Jamaica
Source: J Health Pollut. 2020 May 4;10(26):200601. doi: 10.5696/2156-9614-10.26.200601 (PMC7269324; doi:10.5696/2156-9614-10.26.200601)
Supplement: Supplementary file 2 [file Ricketts_Supplemental2.docx]

**Supplemental Material 2**

| **Physical Description of Skin Lightening Products** | | | | |
| --- | --- | --- | --- | --- |
| **Sample ID** | **Commercial Product name** | **Advertised purpose** | **Country of Origin** | **Physical Description** |
| SLP001 | 7-Day Magic Lightening Cream |  | Unknown | White, cream |
| SLP002 | African Formula Skin Lightening Cream | Gradual fading of dark (brownish) areas in the skin such as freckles, age and liver spots | EU | Pink, cream |
| SLP003 | Ambi Skincare | Gradually fades dark areas for even, natural skin tone. | Unknown | White, cream |
| SLP004 | Ansep Soap | Keeps skin clear, clean and free from spots, kills surface germs | Indonesia | Dark green, solid |
| SLP005 | Bio Claire Cream | Stimulates lightening process, makes complexion even and clear | Ivory Coast | White, cream |
| SLP006 | Bio Claire Lotion | Stimulates the lightening process in a natural way, ensures complete elimination of cutaneous defects and makes your complexion clear and even, with no mess. | Ivory Coast | White, lotion |
| SLP007 | Bio Claire Oil | Stimulates lightening process, makes complexion even and clear | Ivory Coast | Brown, oil |
| SLP008 | Bio Claire Soap | Stimulates lightening process, makes complexion even and clear | Ivory Coast | Brown, soap bar |
| SLP009 | BioTone | Moisturizes skin, evens skin tone, improves clarity, radiance and soften and smoothes skin. | Lebanon | Dark brown, cream |
| SLP010 | Caro Bright Fast Action | Deeply nourishes and moisturizes skin while working to even out skin tone, improving clarity and radiance. Promotes softer and smoother skin | India | White, cream |
| SLP011 | Caro White Intensive Care | Makes skin light, manageable and smooth | Ivory Coast | Peach, cream |
| SLP012 | Carotis Lightening Body Lotion | Promotes clearer, visibly brighter and lighter skin | Unknown | Dark brown, cream |
| SLP013 | Crusader Soap | Helps prevent skin infections, pimples, boils, prickly heat, lice in the hair | UK | Blue, soap bar |
| SLP014 | Dermo-Gel |  | Unknown | Transparent, gel |
| SLP015 | DermoPlus Exfoliating and Lightening Soap | Nourishes and moisturizes skin | Morocco | Light brown, solid |
| SLP016 | Doctor Clear | Acts efficiently on sensitive skin | Ivory Coast | Light orange, solid |
| SLP017 | Dolly Antiseptic Soap | Prevention and treatment of skin | Lebanon | Blue, solid |
| SLP018 | Fair & Lovely Savon | Cleans your body and eliminates dead cells, gentle exfoliating microbeads | Lebanon | Dark pink, solid |
| SLP019 | Fair & White Antiseptic Soap | Purifies and cleans the skin, skin is left feeling smooth, even and radiant | Lebanon | Brown, solid |
| SLP020 | G&G Dynamiclair Cream | Lightens the skin, reduces brown spots | Unknown | White, cream |
| SLP021 | Glow and White | Prepares the application of the cream or body milk glow & white while eliminating dead cells and impurities | Unknown | White, cream |
| SLP022 | Haloderm Cream |  | Unknown | White, cream |
| SLP023 | Hyprogel |  | Unknown | Light pink, cream |
| SLP024 | Idole Lotion | Gradual fading of dark (brownish) areas in the skin such as freckles, age and liver spots | Unknown | White, cream |
| SLP025 | Idole Soap | Accelerates the elimination of dead cells and combat pigmentary blemishes, deep cleanses the epidermis, lightens complexion and leaves skin smooth and radiant. | EU (Spain) | Brown, solid |
| SLP026 | Immediat Claire | Clarifies, unifies and rids skin of brown spots | Ivory Coast | Yellow, cream |
| SLP027 | KomeFast Super Toning Cream | Tones dark spots and lightens and brightens skin | Jamaica | White, cream |
| SLP028 | La Bamakoise Tamarin Lait Extra Tonique (lotion) | Hydrates, cleans, lightens/removes dark spots and unifies staunch | Ivory Coast | White, lotion |
| SLP029 | L'abidjanaise | Active against acute dermatitis: eczema, psoriasis, prurigo, local inflammatory and allergic reactions, stimulates cutaneous system to bring skin back to its normal condition | Italy | White, cream |
| SLP030 | Lemonvate | Cream reduces appearance of dark brown spots, evening out skin tone | India | Cream, cream |
| SLP031 | Maxi Light | - | Unknown | Peach, cream |
| SLP032 | Metasol Medicated Cream | Fading of dark (brownish) areas in the skin, contains sunscreen to prevent future darkening | EU (Switzerland) | Pink, cream |
| SLP033 | Milk Protein | - | Unknown | Brown, cream |
| SLP034 | Natural Papaya Cream | Active against acute dermatitis: eczema, psoriasis, prurigo, local inflammatory and allergic reactions, stimulates cutaneous system to bring skin back to its normal condition | Italy | White, cream |
| SLP035 | Neoplus Cream Fort | Eliminate dark spots & reduce callus skin | Unknown | White, cream |
| SLP036 | Neoplus Soap Fort | Clear and even skin tone | Unknown | Dark orange, solid |
| SLP037 | Neoprosone Forte Savon |  | Lebanon | Dark blue, solid |
| SLP038 | Neoprosone Gel |  | Unknown | Transparent, gel |
| SLP039 | New Light | Reduces callosity of the skin, smooth down rough skin, renew surface layers and eliminates dead cells | Italy | White, cream |
| SLP040 | Olay Regenerist Lotion |  | Unknown | White, cream |
| SLP041 | Omic Gel | Quickly absorbed, brightening dull and uneven skin tone, promoting luminous complexion | Unknown | Transparent, gel |
| SLP042 | Palmer’s Skin Success | Corrects discolourations such as dark & age spots, post acne scars, uneven skin tone | USA | Light brown, cream |
| SLP043 | Peter Thomas Roth Massaging Bar for Bath | Leaves skin clean, soft and refreshed |  | White, solid |
| SLP044 | Peter Thomas Roth Moisture Infusion | Gently cleanses the skin while delivering moisture |  | White, solid |
| SLP045 | PureSoap | Moisturize and cleanse skin naturally | Trinidad & Tobago | Cream, solid |
| SLP046 | Radiant Skin Lightening Pills | Bleaching | Unknown | Brown |
| SLP047 | Septol Marque Depose | Helps promote healthy skin | England | Dark green, solid |
| SLP048 | Silken | Fades dark spots and freckles, lightens and brightens skin, reduces oiliness | Jamaica | Pearl white, cream |
| SLP049 | Symba | Lightens uneven and dark discoloration, fades freckles, age and liver spots, moisturizes skin | England | Pink, cream |
| SLP050 | Tamarind Lightening Cream | - | Unknown | Brown, cream |
| SLP051 | Tamarind Soap | - | Lebanon | Pink, solid |
| SLP052 | Topiclear Number One Personal Hygiene Soap | Cleanses the skin of impurities, leaves skin fresh without over drying | EU (England) | Light brown, solid |
| SLP053 | Topsomol Oligo Brightening | - | Unknown | Transparent, gel |
| SLP054 | Triple Antibiotic Ointment |  | Unknown |  |
| SLP055 | Ultra Bright Cleansing Bar | Helps lighten the skin, eliminates bacteria that causes pimples dark spots, blackheads and other skin blemishes | India | Light pink, solid |
| SLP056 | Ultra Cream | Gradual fading of dark (brownish) areas in the skin such as freckles, age and liver spots | Unknown | Pearl white, cream |
| SLP057 | Ultra Bright Brightening Gel | Nourishes & moisturizes skin, evens out skin tone | India | Transparent, gel |
| SLP058 | Virginity Soap | Cleansing the most sensitive area of women without leaving any residue, maintains proper moisture of skin, protects irritations and bacterial infections, tightens the vaginal muscle | China | Orange, solid |
| SLP059 | White cream | Bleaching | Unknown | White, cream |
| SLP060 | Yellow cream | Bleaching | Unknown | Yellow, cream |
