# Supplementary material for: Mercury Exposure Associated with Use of Skin Lightening Products in Jamaica
Source: J Health Pollut. 2020 May 4;10(26):200601. doi: 10.5696/2156-9614-10.26.200601 (PMC7269324; doi:10.5696/2156-9614-10.26.200601)
Supplement: Supplementary file 3 [file Ricketts_Supplemental3.docx]

**Supplemental Material 3**

| **Mercury Concentrations in Products** | | | |
| --- | --- | --- | --- |
| **Sample ID** | **Product name** | **Mercury concentration/ ppm** | |
|  |  | **XRF** | **CVAAS** |
| SLP01 | 7-Day Magic Lightening Cream | < LOD | 0.16 |
| SLP02 | African Formula Skin Lightening Cream | < LOD | 0.33 |
| SLP03 | Ambi Skincare | < LOD |  |
| SLP04 | Ansep Soap | < LOD |  |
| SLP05 | Bio Claire Cream | < LOD | 0.72 |
| SLP06 | Bio Claire Lotion | < LOD | 0.12 |
| SLP07 | Bio Claire Oil | < LOD | 0.06 |
| SLP08 | Bio Claire Soap | < LOD | 0.05 |
| SLP09 | BioTone | < LOD | 0.10 |
| SLP10 | Caro Bright Fast Action | < LOD | 0.05 |
| SLP11 | Caro White Intensive Care | < LOD | 0.12 |
| SLP12 | Carotis Lightening Body Lotion | < LOD |  |
| SLP13 | Crusader Soap | < LOD | 0.06 |
| SLP14 | Dermo-Gel | < LOD |  |
| SLP15 | DermoPlus Exfoliating and Lightening Soap | < LOD |  |
| SLP16 | Doctor Clear | < LOD |  |
| SLP17 | Dolly Antiseptic Soap | < LOD | 0.45 |
| SLP18 | Fair & Lovely Savon | < LOD |  |
| SLP19 | Fair & White Antiseptic Soap | < LOD |  |
| SLP20 | G&G Dynamiclair Cream | < LOD | 0.29 |
| SLP21 | Glow and White | < LOD | 0.06 |
| SLP22 | Haloderm Cream | < LOD | 2.17 |
| SLP23 | Hyprogel | < LOD | 0.05 |
| SLP24 | Idole Lotion | < LOD |  |
| SLP25 | Idole Soap | < LOD | 0.15 |
| SLP26 | Immediat Claire | < LOD |  |
| SLP27 | KomeFast Super Toning Cream | < LOD | 4.10 |
| SLP28 | La Bamakoise Tamarin Lait Extra Tonique (lotion) | < LOD | 0.10 |
| SLP29 | L'abidjanaise | < LOD |  |
| SLP30 | Lemonvate | < LOD |  |
| SLP31 | Maxi Light | < LOD | 1.12 |
| SLP32 | Metasol Medicated Cream | < LOD | 0.19 |
| SLP33 | Milk Protein | < LOD |  |
| SLP34 | Natural Papaya Cream | < LOD | 0.26 |
| SLP35 | Neoplus Cream Fort | < LOD |  |
| SLP36 | Neoplus Soap Fort | < LOD | 0.15 |
| SLP37 | Neoprosone Forte Savon | < LOD |  |
| SLP38 | Neoprosone Gel | < LOD |  |
| SLP39 | New Light | < LOD |  |
| SLP40 | Olay Regenerist Lotion | < LOD |  |
| SLP41 | Omic Gel | < LOD |  |
| SLP42 | Palmer’s Skin Success | < LOD |  |
| SLP43 | Peter Thomas Roth Massaging Bar for Bath | < LOD |  |
| SLP44 | Peter Thomas Roth Moisture Infusion | < LOD |  |
| SLP45 | PureSoap | < LOD |  |
| SLP46 | Radiant Skin Lightening Pills | < LOD | 0.05 |
| SLP47 | Septol Marque Depose | < LOD |  |
| SLP48 | Silken | 17547.16 |  |
| SLP49 | Symba | < LOD |  |
| SLP50 | Tamarind Lightening Cream | < LOD | 0.29 |
| SLP51 | Tamarind Soap | < LOD |  |
| SLP52 | Topiclear Number One Personal Hygiene Soap | < LOD |  |
| SLP53 | Topsomol Oligo Brightening | < LOD |  |
| SLP54 | Triple Antibiotic Ointment | < LOD |  |
| SLP55 | Ultra Bright Cleansing Bar | < LOD | 0.18 |
| SLP56 | Ultra Cream | < LOD |  |
| SLP57 | Ultra Bright Brightening Gel | < LOD |  |
| SLP58 | Virginity Soap | < LOD |  |
| SLP59 | White cream | 465.73 |  |
| SLP60 | Yellow cream | 422.04 |  |
